# Cost-effectiveness analysis of liver transplantation in biliary atresia according to the severity of end-stage liver disease

**DOI:** 10.1186/s12887-023-04270-0

**Published:** 2023-09-02

**Authors:** Boonyanurak Sihaklang, Songpon Getsuwan, Oraluck Pattanaprateep, Napapat Butsriphum, Chatmanee Lertudomphonwanit, Pornthep Tanpowpong, Chollasak Thirapattaraphan, Suporn Treepongkaruna

**Affiliations:** 1 Division of Gastroenterology, Department of Pediatrics, Faculty of Medicine Ramathibodi Hospital, Mahidol University, 270 Rama VI Road, Thung Phayathai, Ratchathewi, Bangkok, Thailand; 2 Department of Pediatrics, Institute of Medicine, Suranaree University of Technology, Nakhon Ratchasima, Thailand; 3 Ramathibodi Excellence Center in Organ Transplantation, Faculty of Medicine Ramathibodi Hospital, Mahidol University, Bangkok, Thailand; 4 Department of Clinical Epidemiology and Biostatistics, Faculty of Medicine Ramathibodi Hospital, Mahidol University, Bangkok, Thailand; 5 Division of Pediatric Surgery, Department of Surgery, Faculty of Medicine Ramathibodi Hospital, Mahidol University, Bangkok, Thailand

**Keywords:** Biliary atresia, Liver cirrhosis, Liver transplantation, Children, Cost-effectiveness analysis, Hospital costs, Mortality

## Abstract

**Background:**

Timing for liver transplantation (LT) in biliary atresia (BA) children with end-stage liver disease (ESLD) is associated with all-cause mortality. The cut-off value of pediatric end-stage liver disease (PELD) score for LT consideration varies across institutions. We aimed to determine the cost-effectiveness of LT to prevent death among BA children registered on the waiting list with different severities of ESLD.

**Methods:**

Subjects were BA children aged < 12 years at a transplant center between 2010 and 2021. A decision tree was developed for cost-effectiveness analysis from a hospital perspective to compare all-cause death between patients initially registered with a low PELD score (< 15) and a high PELD score (≥ 15). Each patient’s direct medical cost was retrieved from the beginning of registration until 5 years after LT, adjusted with an inflation rate to 2022 Thai Baht (THB).

**Results:**

Among 176 children, 138 (78.4%) were initially registered with the high PELD score. The cost and mortality rate of the low PELD score group (THB1,413,424 or USD41,904 per patient and 31.6% mortality) were less than the high PELD score group (THB1,781,180 or USD52,807 per patient and 47.9% mortality), demonstrating the incremental cost-effectiveness ratio (ICER) of THB2,259,717 or USD66,994 per death prevented. The cost of early post-operative admission had the highest effect on the ICER. Considering the break-even analysis, cost among children initially registered at the low PELD score was also less expensive over time.

**Conclusions:**

Registration for LT at PELD score < 15 was more cost-effective to prevent death among BA children with ESLD.

**Supplementary Information:**

The online version contains supplementary material available at 10.1186/s12887-023-04270-0.

## Introduction

Biliary atresia (BA) is the most common cause of neonatal cholestasis, which could lead to end-stage liver disease (ESLD) in children [1]. BA Children with delayed diagnosis or failed hepatic portoenterostomy (HPE) generally develop several complications of ESLD [2], requiring both emergency and long-term management, utilizing public health care resources, and having financial burdens to the public health system.

Liver transplantation (LT) is a life-saving procedure with a high successful rate in BA with ESLD [3]. The Thai Pediatric LT registry has reported a 90-day and 1-year survival rates of 93.7% and 90.5%, respectively [4]. Although children who underwent LT have a satisfactory health-related quality of life, they need lifelong immunosuppressive therapy [5]. Some LT children may suffer from post-LT complications, including graft rejection, vascular and bile duct complications, opportunistic infection, and adverse effects from immunosuppressive therapy [3, 6].

Common indications for LT are liver deterioration, particularly decompensated cirrhosis, and complications of ESLD [7–9]. Finding an optimal timing to perform LT can be challenging. ‘Delayed’ LT can affect children's health, as they may have serious sequelae of the disease or die before the operation. In contrast, children who undergo ‘early’ LT could receive a longer duration of high-cost immunosuppressive therapy and a greater risk of its adverse effects.

The United Network for Organ Sharing (UNOS) has developed Pediatric End-stage Liver Disease (PELD) score to prioritize pre-LT candidates aged < 12 years by using the following data: age, growth status, serum albumin, total bilirubin and international normalized ratio [10]. Patients with a higher PELD score have a greater rate of pre-LT mortality [11]. According to our preliminary study, which has not been published, PELD score ≥ 15 was associated with an increase of pre-LT mortality, similar to a finding from de Vries et al. [12] Moreover, Arnon et al. [13] have found that LT for moderate liver disease has increased expected life-years and survival rates in BA children when compared to LT for severe disease. Hence, early registration before the severe disease may have some benefits among these children. Unfortunately, the cut-off value of PELD score for LT registration varies among health care institutions [11, 14].

LT is associated with high health care expenditures [15]. A previous study has mentioned that a higher PELD score is related to greater health care cost in the first year after LT [16]. To our knowledge, no cost-effectiveness analysis has been performed to evaluate an optimal disease severity for LT registration. Even though most of health care expenditures for pediatric LT can be reimbursed from Thailand’s Universal Coverage Scheme (UCS) [4], many LT centers must absorb any cost which exceeds the government’s reference price. From a transplant center perspective, we aimed to study the cost-effectiveness of LT which could prevent all-cause death among BA children registered on the waiting list with different severity of ESLD. The secondary objective was to estimate the break-even point of LT among children on the waiting list with different severity of ESLD.

## Methods

### Study population

A cost-effectiveness study from a hospital perspective was conducted at an active LT center in Bangkok, one of the major referral centers in Thailand. Enrolled subjects were BA children aged 6 months to 12 years who were diagnosed with ESLD and registered on the LT waiting list from January 2010 to December 2021. The severity of ESLD was evaluated by PELD score, which could be calculated from the following equation: [10] PELD = 0.436 × [age (< 1 year)] – 0.687 × Log_e_ (albumin g/dL) + 0.480 × Log_e_ (total bilirubin mg/dL) + 1.87 × Log_e_ (international normalized ratio) + 0.667 (growth failure (< –2 standard deviation present). The immunosuppressive protocol for LT was consistent during the study period, mainly comprising corticosteroids, tacrolimus, and mycophenolate mofetil. Patients with a plan to undergo ABO-incompatible LT were excluded. The participants were divided into two groups according to their PELD scores at the initial registration: low PELD score (< 15) and high PELD score (≥ 15).

### Data collection

Clinical information was retrospectively collected from the medical record, including the demographic data, all-cause death, and complications before and after LT.

This study focused on direct medical cost in LT recipients from the beginning of registration until 5 years after LT. The cost was retrieved from the Finance Department at our institution and adjusted with an inflation rate to 2022 Thai Baht (THB) according to the data from the Bank of Thailand (1 USD = 33.73 THB) [17]. The cost concerning LT can be divided into three periods as follow:

1. *Cost in pre-LT period*, starting from the registration until before LT operation. The cost was presented as cost in the outpatient department (OPD) and cost in the inpatient department (IPD). The cost in this phase included cost in a pre-LT evaluation and cost of treatment regarding the complications of ESLD.
2. *Cost in LT period*, starting from the LT operation until the end of early post-operative period, which was less than 90 days after LT. The main cost in this phase was in the early post-operative admission.
3. *Cost in post-LT period*, starting from 90 days after LT until 5 years after LT. The cost was demonstrated in the following categories: drugs, laboratory investigations, radiologic investigations, and other cost (medical fee, medical equipment, room charge, etc.).

### Cost-effectiveness analysis

**Fig. 1:**
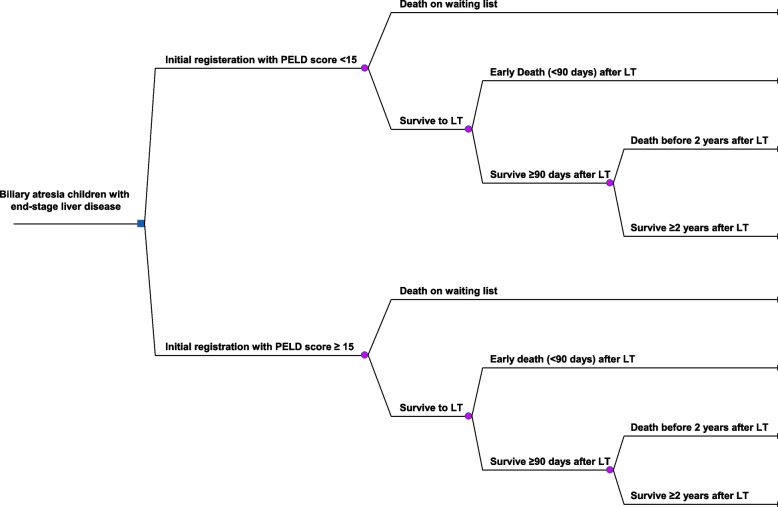
Decision tree model for biliary atresia children with end-stage liver disease. The decision tree model shows the management strategies among biliary atresia children with end-stage liver disease in the study. The rectangular represents the decision to either initially register for liver transplantation (LT) at Pediatric End-stage Liver Disease (PELD) score < 15 or at PELD score ≥ 15. Chance nodes are demonstrated as circles. Some children may die before LT while others may survive and undergo LT. The interested outcome was the all-cause mortality among children registered for LT. LT: liver transplantation; PELD score: Pediatric End-stage Liver Disease score

Cost-effective analysis in a hospital perspective was based on a decision tree model, using Plant-A-Tree program (International Decision Support Initiative Version 1.0, UK). Our model was constructed to compare between the decisions to initially register BA children with ESLD at the low PELD score (< 15) and at the high PELD score (≥ 15) (Fig. 1). The interested outcome of effectiveness was the reduction of all-cause mortality. The incremental cost-effectiveness ratio (ICER) was defined by the difference in total cost (Cost _low PELD score_ – Cost _high PELD score_) divided by the difference in effectiveness (Mortality rate _low PELD score_ – Mortality rate _high PELD score_) of the two decisions. We determined potential factors affecting ICER by applying one-way sensitivity analysis from a tornado diagram (TreePlan Version 1.61, San Francisco, CA). In addition, break-even analysis was studied from the cumulative direct cost over time.

### Statistical analyses

Data were analyzed by using STATA program (StataCorp. Version 14, College Station, TX). Comparisons between the low PELD score and the high PELD score groups were examined by using chi-squared test and Fisher’s exact test for categorical variables with normal and non-normal distribution, respectively. Similarly, Student’s t-test and Mann–Whitney U test were applied for continuous variables. *P* < 0.05 was considered statistically significant.

### Ethical consideration

This study followed the Helsinki declaration and was approved by the Human Research Ethics Committee at our institution.

## Results

### Patient characteristics

A total of 176 patients were initially registered for LT at the average (SD) PELD score of 20.4 (8.3), consisting of 38 patients (21.6%) registered at the low PELD score and 138 patients (78.4%) registered at the high PELD score with the average (SD) score at the registration of 11 (2.7) and 23.1 (5.3), respectively. A higher percentage of patients in the low PELD score group underwent HPE (84.2% *vs* 58%, *P* = 0.02). Complications of ESLD and duration of IPD admission before LT were similar between the two groups. The numbers of children who underwent LT with PELD score-exception conditions, including hepatorenal syndrome, hepatopulmonary syndrome, and refractory gastrointestinal bleeding, were not significantly different between both groups. Most children registered for LT were under the UCS; only approximately 5% were under the government or state enterprise officer scheme. Table 1 summarizes the characteristics of the participants.

**Table 1:** Characteristics of biliary atresia children with end-stage liver disease who were registered for liver transplantation (*N* = 176)

**Characteristics**	**Low** **PELD score group**^**a**^** (*N* = 38)**	**High** **PELD score group**^**b**^** (*N* = 138)**	***P***
Gender: Male, *N* (%)	19 (50.0)	57 (41.3)	0.5
Underwent hepatic portoenterostomy, *N* (%)	32 (84.2)	80 (58.0)	0.02
Total duration of any admissions before liver transplantation (days), median (IQR)	15 (5, 97)	39 (15, 52)	0.4
Complications of end-stage liver disease before liver transplantation, *N* (%)			
- Upper gastrointestinal bleeding	15 (39.5)	65 (47.1)	0.4
- Sepsis	12 (31.6)	65 (47.1)	0.08
- Spontaneous bacterial peritonitis	6 (15.8)	38 (27.5)	0.1
PELD score at the registration, mean (SD)	11 (2.7)	23.1 (5.3)	< 0.01
Age at the registration (months), median (IQR)	11.5 (7, 21)	7 (6, 10)	< 0.01
Total bilirubin at the registration (mg/dL), mean (SD)	14 (6.9)	16.8 (7.2)	0.04
Serum albumin at the registration (g/dL), mean (SD)	2.6 (0.6)	2.4 (0.6)	0.06
International normalized ratio at the registration, mean (SD)	1.2 (0.2)	1.4 (0.3)	< 0.01
Patients with PELD score-exception conditions requiring LT^c^, *N* (%)	3 (7.9)	6 (4.3)	0.41
Health insurance: Universal Coverage Scheme, *N* (%)	36 (94.7)	130 (94.2)	> 0.99

*PELD score* Pediatric End-stage Liver Disease score

^a^PELD score < 15

^b^PELD score ≥ 15

^c^E.g., hepatopulmonary syndrome, hepatorenal syndrome, refractory gastrointestinal bleeding

### Clinical outcomes

During the study period, a total of 78 children (44.3%) registered for LT died, consisting of 70 children before LT, and 8 children after LT. The overall mortality rate was 31.6% in the low PELD score group and 47.9% in the high PELD score group. During pre-LT period, patients in the low PELD score group had a lower mortality rate (26.3% *vs* 43.5%, *P* = 0.05). The most common cause of death before LT was sepsis (50%), followed by upper gastrointestinal bleeding (20%). Patients who did not undergo HPE had a higher PELD score at the registration (23.1 *vs* 18.8, *P* < 0.01) with a higher mortality rate on the waiting list (50% *vs* 44.5%, *P* = 0.04) and a younger age at LT (1.1 years *vs* 1.6 years, *P* < 0.01) when compared to patients who underwent the operation (Supplementary Table 1).

Children who survived beyond the pre-LT period mostly underwent living donor LT, which was comparable between both groups (*P* = 0.1). The mean (SD) PELD score at LT was 10.8 (2.8) in the low PELD score group and 21.5 (3.9) in the high PELD score group. None of the children who were initially registered with the low PELD score had PELD score ≥ 15 at LT. Patients in the low PELD score group had a longer waiting time (14.5 months, IQR: 12.4, 24.5) when compared to the high PELD score group (7 months, IQR: 5.3, 10.4) (*P* < 0.01) with an older age at LT (2.2 years, IQR: 1.5, 3.5 *vs* 1.2 years, IQR: 1, 1.8) (*P* = 0.03).

After LT, patients in the low PELD score group had a shorter duration of early post-operative admission (38 days, IQR: 32, 49) than the high PELD score group (49 days, IQR: 32, 79) (*P* = 0.03). The mortality, complications, and re-transplant rate after LT were not statistically different between the two groups. Most death after LT occurred in the early post-operative period. Two patients died after 90 days post-LT due to post-transplant lymphoproliferative disorders. One patient required re-LT due to hepatic artery thrombosis. Table 2 describes the outcomes of participants in the study.

**Table 2:** Outcomes of biliary atresia children with end-stage liver disease who were registered for liver transplantation (*N* = 176)

**Characteristics**	**Low** **PELD score group**^**a**^	**High** **PELD score group**^**b**^	***P***
**Children who died on the waiting list before liver transplantation (*N* = 70)**	***N* = 10**	***N* = 60**	
Death age (years), median (IQR)	1.5 (0.9, 3.3)	1.1 (0.8, 1.8)	0.3
Duration from registration to death (months), median (IQR)	4.4 (1.8, 10.8)	3.7 (1.7, 8.1)	0.7
*Causes of death, N (%)*			
- Sepsis	5 (50)	30 (50)	> 0.99
- Upper gastrointestinal bleeding	2 (20)	12 (20)	> 0.99
- Intracranial hemorrhage with brain herniation	2 (20)	5 (8.3)	0.2
- Others	1 (10)	8 (13.3)	> 0.99
- Unknown	0	5 (8.3)	0.7
**Children who underwent liver transplantation (*N* = 106)**	***N* = 28**	***N* = 78**	***P***
Age at liver transplantation (years), median (IQR)	2.2 (1.5, 3.5)	1.2 (1, 1.8)	< 0.01
Living donor liver transplantation, *N* (%)	25 (89.3)	76 (97.4)	0.1
PELD score at transplantation, mean (SD)	10.8 (2.8)	21.5 (3.9)	< 0.01
Waiting time for liver transplantation (months), median (IQR)	14.5 (12.4, 24.5)	7 (5.3, 10.4)	0.03
Duration of inpatient admission during transplant operation, (days), median (IQR)	38 (32, 49)	49 (32, 79)	0.03
Duration of intensive care units (ICU) stay after the operation (days), median (IQR)	7.5 (5, 14)	8 (5, 15)	0.5
Death after liver transplantation, *N* (%)	2 (7.1)	6 (7.7)	0.9
*Complications after liver transplantation, N (%)*			
- Re-liver transplantation	0	1 (1.3)	0.5
- Bile duct complications	12 (42.9)	23 (29.5)	0.2
- Vascular complications	11 (39.3)	28 (35.9)	0.3
- Acute cellular rejection	12 (42.9)	23 (29.5)	0.2
- Culture-proven bacterial infection	15 (53.6)	48 (61.5)	0.4
- Cytomegalovirus infection	12 (42.9)	31 (39.7)	0.8
- Cytomegalovirus disease	1 (3.6)	6 (7.7)	0.4
- Epstein-Barr virus infection	2 (7.1)	8 (10.3)	0.5
- Post-transplant lymphoproliferative disorders	1 (3.6)	7 (9.0)	0.2

*PELD score* Pediatric End-stage Liver Disease score

^a^PELD score < 15

^b^PELD score ≥ 15

### Direct medical cost

In the low PELD score group, direct medical cost during IPD admission in the pre-LT period (28,394 THB, IQR: 5,016, 66,980) and in the early post-operative period (730,398 THB, IQR: 604,467, 1,126,810) were less than the high PELD score group (55,751 THB, IQR: 16,487, 174,989, and 863,488 THB, IQR: 701,843, 1,325,659, respectively) (*P* = 0.02 and* P* = 0.03, respectively). However, direct medical cost in the OPD was higher in the low PELD score group (43,594 THB *vs* 23,669 THB, *P* = 0.01). In the post-LT period, the total direct medical cost of both two groups tended to decrease over time (Table 3). According to the cost categories, cost of drugs was the highest in the first year after LT. In addition, the cost of the laboratory investigations within 90 days to 2 years after LT among the low PELD score group (81,184 THB, IQR: 60,930, 117,436) was less than the high PELD score group (117,474 THB, IQR: 73,883, 179,488) (*P* = 0.03).

**Table 3:** Direct medical cost per patient among biliary atresia children with end-stage liver disease who were registered for liver transplantation (*N* = 176)

**Cost (Thai Baht per patient)**	**Low PELD score group**^**a**^** (*N* = 38)**	**High PELD score group**^**a**^** (*N* = 138)**	***P***
**Pre-liver transplant period, median (IQR)**			
- Inpatient department	28,394 (5,016, 66,980)	55,751 (16,487, 174,989)	0.02
- Outpatient department	43,594 (18,303, 78,455)	23,669 (12,962, 436,189)	0.01
**Liver transplant period, median (IQR)**			
- Early post-operative period or within 90 days after transplant	730,398 (604,467, 1,126,810)	863,488 (701,843, 1,325,659)	0.03
**Post-liver transplant period, median (IQR)**			
*90 days to 2 years after liver transplantation*			
- Total cost	642,854 (475,624, 831,861)	715,728 (516,999, 912,077)	0.4
- Drugs	279,959 (191,239, 401,882)	303,208 (228,915, 397,220)	0.3
- Laboratory investigations	81,184 (60,930, 117,436)	117,474 (73,883, 179,488)	0.03
- Radiologic investigations	10,675 (4,413, 20,130)	12,479 (6,744, 36,511)	0.2
- Others^a^	245,157 (129,750, 324,774)	243,078 (131,738, 385,688)	0.7
*2 to 3 years after liver transplantation*			
- Total cost	222,448 (183,550, 304,332)	235,037 (156,175, 296,810)	0.7
- Drugs	161,580 (125,853, 262,407)	142,036 (116,421, 213,169)	0.4
- Laboratory investigations	23,325 (19,385, 52,042)	29,082 (20,287, 52,101)	0.4
- Radiologic investigations	3,229 (2,212, 8,187)	4,045 (2,222, 10,222)	0.5
- Others^a^	20,430 (13,585, 47,933)	9,546 (1,441, 33,122)	0.02
*3 to 4 years after liver transplantation*			
- Total cost	157,840 (135,552, 211,021)	166,488 (128,897, 231,206)	0.6
- Drugs	117,978 (99,398, 150,127)	135,890 (107,506, 172,831)	0.4
- Laboratory investigations	16,409 (8,940, 28,337)	19,330 (13,330, 37,287)	0.2
- Radiologic investigations	2,229 (0, 3,651)	2,219 (221, 6,258)	0.8
- Others^a^	13,096 (6,870, 21,390)	3,075 (761, 23,523)	0.01
*4 to 5 years after liver transplantation*			
- Total cost	161,561 (107,273, 250,702)	151,101 (112,984, 272,755)	0.7
- Drugs	127,489 (79,740, 186,027)	118,842 (98,267, 178,172)	0.8
- Laboratory investigations	13,185 (10,244, 28,814)	17,528 (12,356, 37,927)	0.2
- Radiologic investigations	2,212 (0, 3,308)	2,216 (0, 3,729)	0.7
- Others^a^	12,288 (8,028, 25,506)	1,304 (385, 15,290)	< 0.01

*PELD score* Pediatric End-stage Liver Disease score

^a^PELD score < 15

^b^PELD score ≥ 15

^c^Cost of medical fee, medical equipment, room charge, etc.

### Cost-effective analysis of LT to prevent death

**Fig. 2:**
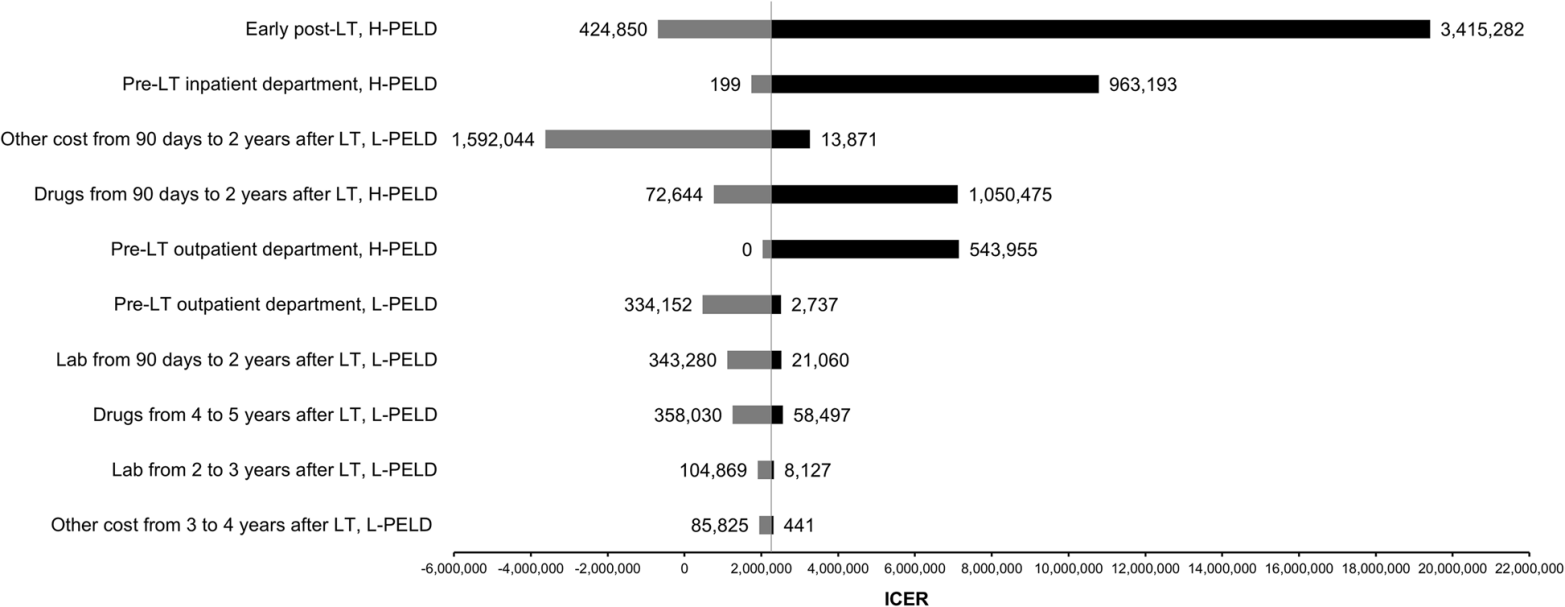
Tornado diagram. A tornado diagram shows incremental cost-effectiveness ratio (ICER) estimates in Thai Baht (THB) per death prevented. The black bars represent the increasing ICER from the reference ICER, whereas the grey bars represent the decreasing ICER. Factors are demonstrated in descending orders according to their effects on the ICER. The most sensitive parameter affecting the ICER was cost in the early post-operative period after liver transplantation (LT), followed by cost in the any admissions before liver transplantation (pre-LT inpatient department) among children initially registered with the high Pediatric End-stage Liver Disease score (H-PELD). H-PELD: high PELD score group; ICER: incremental cost-effectiveness ratio; L-PELD: low PELD score group; Other cost: cost in medical fee, medical equipment, room charge, etc.; LT: liver transplantation; PELD score: Pediatric End-stage Liver Disease score

According to the decision tree model, the total direct cost of the low PELD score and the high PELD score group were 1,413,424 THB and 1,781,180 THB, respectively, demonstrating the ICER of 2,259,717 THB per death prevented. One-way sensitivity analysis from the tornado diagram (Fig. 2) revealed that the cost in the early post-operative period was the most sensitive parameters which could alter the ICER, followed by the cost of IPD admission in the pre-LT period among children with the high PELD score.

**Fig. 3:**
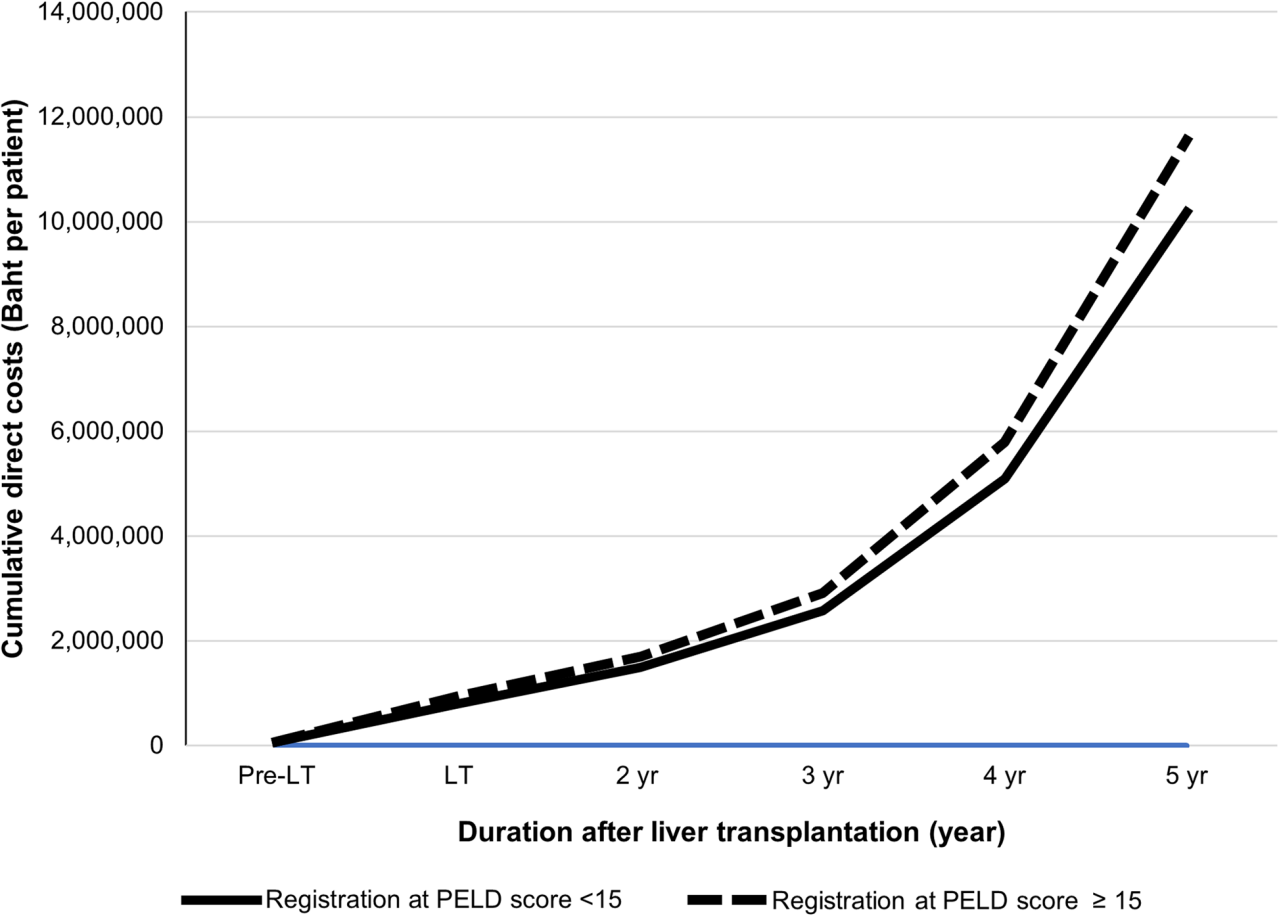
Break-even analysis. A graph illustrates the cumulative direct medical cost among biliary atresia children registered for liver transplantation (LT) over time. The x-axis is the duration starting from registration to 5 years (yr) after LT. The y-axis is the cumulative direct cost in Thai Baht (THB) per patient. The solid line represents the cost in children initially registered at Pediatric End-stage Liver Disease (PELD) score < 15, and the dashed line represents the cost in children initially registered at PELD score ≥ 15. According to the graph, the cumulative cost was not only less in the lower PELD score group at the beginning of the registration, but also over time. LT: liver transplantation; PELD score: Pediatric End-stage Liver Disease score; yr: year

To illustrate the break-even analysis, a comparison of the cumulative cost over time between both groups was presented in Fig. 3. We observed that the cost of the low PELD score group was not only less expensive at the beginning, but also over time.

## Discussion

According to our findings, initial registration for LT at PELD score < 15 had lower all-cause mortality and less total direct medical cost when compared to registration at a higher PELD score, suggesting that early registration was more cost-effective to prevent death in BA children with ESLD. Certain direct medical cost was different among children registered with the low and with the high PELD scores in each period of LT. Moreover, the cumulative cost of initial registration with the low PELD score was not only less at the beginning, but also over a 5-year period.

Previous studies have examined the relationship between PELD score and mortality in the pre-LT and post-LT periods. According to a large study in children with chronic liver disease from the UNOS [11], PELD score is related to 90-day pre-LT mortality, similar to our findings. While a study reported that children underwent LT at a lower PELD score have less mortality after LT [18], the association is not significant according to Bourdeaux et al. [19], comparable to our finding in the post-LT period. Nevertheless, the number of post-LT death was quite low in this study, which could limit the robustness of statistical analysis.

Since children registered with the low PELD score had less all-cause mortality which was calculated from both pre- and post-LT periods, early registration for LT may be beneficial in BA children with ESLD. As the majority of our children already had high PELD score at the referral and thus were initially registered with the high score, this may emphasize the necessity of earlier referral in BA children, which should be addressed in those with inadequate clearance of jaundice at the age of 6 to 9 months [7].

This study revealed that the total direct cost was less in the low PELD score group. In the pre-LT period, the cost during IPD admission was also significantly lower in the low PELD score group, although the duration of IPD admission was not statistically different. Unfortunately, we had limited information regarding the details of the cost in this period. We also observed that the cost in the OPD was higher in the group with the low PELD score, which may be contributed by a longer duration of follow-up through the OPD visits. Patients in the low PELD score group were older at LT in our study, probably due to the slower progression of the disease. A larger proportion of children in this group underwent HPE, which could delay the necessity for LT in several reports [20–22], even in patients who underwent the operation at age > 60 days [23]. The waiting time for LT in the low PELD score group was longer than the high PELD score group. This was due to the policy of our center to prioritize pre-LT evaluation for donors of patients with a very high PELD score (> 20) at the registration, as well as the LT operation, to minimize the waiting list mortality.

The cost of LT is usually peak within 3 months after LT [24], which was defined as the LT-period in this study. From our tornado diagram, the cost of the high PELD score group was the most sensitive parameters affecting the ICER. Our study found that the low PELD score group had less cost in this period (730,398 THB *vs *863,488 THB). The cost in both groups was higher than the National Health Security Office’s reference price that the hospital could reimburse under UCS, which was the major health insurance scheme in this study (almost 95% of the participants). Currently, the reference price is approximately 470,000–625,000 THB during this period, consisting of 410,000–565,000 THB for the admission during the transplant operation and 60,000 THB for the outpatient care within 3 months after LT. Nevertheless, the cost of LT in this study was lower than the reported cost in North America [24, 25].

Associated factors with the cost of LT have been reported in previous studies. The cost is known to be correlated with the duration of admission during the LT operation and peri-operative care [26], which was significantly less among the low PELD score group in this study. While one study described that living donor LT had more cost within 1 year after LT when compared to deceased donor LT [16], the difference is not demonstrated in another study [26]. Nevertheless, the donor types were not significantly different between the groups of our children.

In post-LT period, the total direct medical cost was peak during the first year after surgery and gradually decreased, similar to previous studies in the US [24] and Canada [25]. Drug cost was the highest among all categories in this study which were mostly contributed by immunosuppressive therapy. According to the study from the US [24], the cost of drugs is the second highest in the OPD visits, followed the cost of laboratory and radiologic investigations. However, the combination of cost in the investigations was less than the cost of drugs in this study. Moreover, we found that the high PELD score group had higher cost of investigation from 90 days to 2 years after LT. Therefore, avoiding over-investigation may be considered in these children.

This study has some limitations. First, the direct medical cost was only retrieved from a single LT center and did not include the cost from other hospitals. Additionally, we did not study indirect cost and quality of life. The application of PELD score also have some limitations in infants [14]; some children may have ESLD-related complications requiring LT, regardless of the PELD score [7]. Nevertheless, the numbers of children with the PELD score-exception conditions were not different between both groups. Moreover, the study was conducted over a long period of time, which details of management may change, although the main protocol for LT was consistent during the study period. In addition, we did not explore direct non-medical cost which could be associated with the number of visits and duration from registration to LT.

## Conclusions

Initial registration for LT at PELD score < 15 was more cost-effective to prevent the mortality among BA children with ESLD. The cumulative cost of initial registration at the low PELD score was not only less at the beginning, but also over time. For this reason, early referral and registration for LT should be emphasized to prevent death in BA children with ESLD.

### Supplementary Information

**Additional file 1: Supplementary Table 1.** Characteristics of biliary atresia children who were registered for liver transplantation according to the history of hepatic portoenterostomy (*N*=176).

## Data Availability

The datasets used and/or analyzed during the current study are available from the corresponding author on reasonable request.

## Abbreviations

BA: Biliary atresia
ESLD: End-stage liver disease
HPE: Hepatic portoenterostomy
ICER: Incremental cost-effectiveness ratio
IPD: Inpatient department
LT: Liver transplantation
OPD: Outpatient department
PELD score: Pediatric End-stage Liver Disease score
THB: Thai Baht
UCS: Universal Coverage Scheme
UNOS: United Network for Organ Sharing
USD: United States Dollars
