# Supplementary material for: Cost-effectiveness analysis of liver transplantation in biliary atresia according to the severity of end-stage liver disease
Source: BMC Pediatr. 2023 Sep 2;23:439. doi: 10.1186/s12887-023-04270-0 (PMC10474723; doi:10.1186/s12887-023-04270-0)
Supplement: Supplementary file 1 — Additional file 1: Supplementary Table 1. Characteristics of biliary atresia children who were registered for liver transplantation according to the history of hepatic portoenterostomy (N=176). [file 12887_2023_4270_MOESM1_ESM.docx]

# Supplementary Table 1 Characteristics of biliary atresia children who were registered for liver transplantation according to the history of hepatic portoenterostomy (N=176)

| **Characteristics** | **Underwent hepatic portoenterostomy (N=112)** | **Did not undergo hepatic portoenterostomy (N=64)** | ***P*** |
| --- | --- | --- | --- |
| PELD score at registration, mean (SD) | 18.8 (6.7) | 23.1 (6.2) | <0.01 |
| Death before liver transplantation, N (%) | 38 (44.5) | 32 (50) | 0.04 |
| Age at liver transplantation* (years), median (IQR) | 1.6 (1.1, 2.4) | 1.1 (0.9, 1.6) | <0.01 |
| Death after liver transplantation*, N (%) | 5 (6.8) | 3 (9.4) | 0.7 |

* N=74 in patients who underwent hepatic portoenterostomy and N=32 in patients who did not undergo the operation

PELD score: Pediatric End-stage Liver Disease score
